# Mitochondrial Stress Orchestrates Tumor Immune Evasion and Immunotherapy Resistance

**DOI:** 10.3390/cells15100890

**Published:** 2026-05-13

**Authors:** Ayhan Bilir, Berna Yıldırım, Mete Hakan Karalök

**Affiliations:** 1 Department of Histology and Embryology, Faculty of Medicine, Istanbul Atlas University, Istanbul 34403, Turkey; berna.yildirim@atlas.edu.tr; 2 Department of Obstetrics and Gynecology, Faculty of Medicine, Istanbul Atlas University, Istanbul 34403, Turkey; hakan.karalok@atlas.edu.tr

**Keywords:** mitochondria, mitochondrial stress, tumor immune evasion, immunotherapy resistance, immune checkpoint blockade, PD-1/PD-L1 axis, mitophagy, tumor microenvironment, metabolic competition, mtDNA–cGAS–STING, 3D tumor models, organoids

## Abstract

**Highlights:**

- Mitochondrial stress functions as a regulatory axis influencing tumor immune evasion and immunotherapy resistance.
- Immune visibility, immune cell fitness, and tumor microenvironment metabolism define three interconnected dimensions of mitochondrial control.
- mtROS, mtDNA–cGAS–STING signaling, and mitophagy integrate metabolic and immune pathways in a context-dependent manner.
- Metabolic competition represents a major constraint on immune checkpoint blockade efficacy in metabolically active tumors.
- Targeting mitochondrial pathways provides a mechanistically grounded strategy to reprogram immunotherapy-resistant tumor phenotypes.

**Abstract:**

Mitochondrial stress has emerged as a key regulator of tumor–immune interactions, extending beyond its classical bioenergetic role to coordinate metabolic adaptation and immune regulation. Rather than merely accompanying tumor progression, mitochondrial dysfunction contributes to immune evasion and resistance to immunotherapy. Here, we propose that mitochondrial stress functions as a unifying axis governing three key determinants of anti-tumor immunity: immune visibility, immune cell fitness, and the metabolic architecture of the tumor microenvironment. Mechanistically, mitochondrial reactive oxygen species, mitochondrial DNA release, and mitophagy modulate antigen presentation and T cell function. We further highlight emerging experimental platforms, including 3D spheroid and organoid systems, that enable physiologically relevant investigation of mitochondria-driven tumor–immune interactions. Together, this perspective provides a mechanistic framework for understanding and targeting resistance to immune checkpoint blockade.

## 1. Introduction

Mitochondrial stress is increasingly recognized as a central regulator that not only governs tumor cell metabolic adaptation but also reshapes immune visibility, immune cell function, and the metabolic organization of the tumor microenvironment (TME) [1]. Recent evidence further indicates that mitochondrial electron transport chain flux and TCA cycle metabolites can directly influence tumor immunogenicity and antigen presentation [1,2]. In this context, mitochondrial stress refers to functional perturbations affecting mitochondrial integrity, including altered electron transport chain activity, redox imbalance, mitochondrial DNA release, and disruption of mitochondrial dynamics. Importantly, these effects may arise in tumor cells, immune cells, or other components of the tumor microenvironment, and their functional consequences are context dependent.

Immune checkpoint inhibitors, particularly those targeting programmed cell death protein 1 (PD-1) and its ligand PD-L1, have transformed cancer therapy by producing durable clinical responses in selected patient populations [3]. However, a substantial proportion of patients exhibit either primary resistance or develop acquired resistance, limiting long-term efficacy [3]. This resistance cannot be fully explained by tumor-intrinsic genetic alterations alone; instead, current models emphasize a multilayered framework in which metabolic and biophysical properties of the TME play a decisive role [4]. Within this context, T cell dysfunction and exhaustion have emerged as central determinants of therapeutic failure [4,5].

The TME establishes a dynamic metabolic environment that governs tumor–immune interactions [6]. Irregular vascularization combined with high oxygen consumption leads to hypoxia, while enhanced glycolysis and poor perfusion result in lactate accumulation and acidosis [6,7]. Under these conditions, essential nutrients such as glucose and amino acids become limiting, creating direct competition between tumor cells and infiltrating immune cells [6,7].

This metabolic competition has direct functional consequences. Increased glucose consumption by tumor cells suppresses mTOR signaling and glycolytic capacity in T cells, leading to reduced interferon-γ (IFN-γ) production [8]. In parallel, glycolytic intermediates can sustain T cell receptor signaling, linking metabolism to immune activation [9]. Importantly, immune checkpoint signaling itself contributes to metabolic reprogramming [10]. PD-1 engagement suppresses glycolysis and amino acid metabolism while promoting fatty acid oxidation (FAO), shifting T cells toward a metabolically restrained state [10].

Within this framework, mitochondria emerge as a central node integrating metabolic and immune signaling networks [2,11]. Beyond ATP production, mitochondria regulate redox balance and coordinate stress responses [2]. The TME has been shown to suppress mitochondrial biogenesis in T cells, leading to metabolic insufficiency and functional impairment in both murine models and human tumor-infiltrating lymphocytes (TILs) [12]. This process is associated with reduced PGC-1α expression, while its restoration partially rescues mitochondrial function and T cell activity [12].

Mitochondrial stress also contributes directly to T cell exhaustion. Hypoxia combined with persistent antigen stimulation induces mitochondrial dysfunction, accelerating exhaustion and suppressing effector functions [13]. More recent studies show that mitochondrial impairment promotes T cell exhaustion through redox imbalance and HIF-1α-mediated reprogramming [14].

Mechanisms of immunotherapy resistance are not restricted to the local microenvironment but may also involve systemic regulation [3]. Tumors capable of suppressing mitochondrial activity in T cells can evade PD-1 blockade by limiting T cell proliferation and metabolic activation through soluble factors [3]. Conversely, pharmacological enhancement of mitochondrial activity has been shown to restore responsiveness to anti-PD-1 therapy, highlighting mitochondria as a therapeutic target [3].

Mitochondrial function also directly influences tumor cell immunogenicity [1]. Modulation of mitochondrial electron flow can enhance antigen presentation independently of IFN-γ signaling by upregulating MHC-I and antigen-processing machinery at transcriptional and epigenetic levels [1]. In addition, emerging evidence indicates that intercellular mitochondrial transfer and accumulation of mitochondrial DNA mutations within TILs may contribute to T cell dysfunction and poor clinical outcomes during immunotherapy [15].

Despite extensive characterization of immune checkpoint pathways, a unifying mechanistic framework explaining why tumors with similar checkpoint profiles exhibit divergent responses to immunotherapy remains lacking. Here, we propose that mitochondrial stress functions as a central integrative axis that resolves this discrepancy. We define this axis through three interconnected dimensions: immune visibility, immune cell fitness, and metabolic architecture of the tumor microenvironment.

These findings support a model in which mitochondrial stress operates as an organelle-centered regulatory axis within the tumor microenvironment. In this review, we propose this conceptual framework and examine how mitochondrial stress contributes to immune evasion and resistance to immune checkpoint blockade (ICB). The following sections examine mitochondrial stress as an immune signaling hub, its role in antigen presentation, immune dysfunction, and mitophagy [2,7]. This perspective is particularly important for explaining variability in responses to ICB among tumors with similar checkpoint expression profiles. Importantly, this framework does not imply that mitochondrial stress uniformly promotes immune evasion. Rather, its functional outcome depends on defined parameters including intensity, duration, subcellular localization, and cell-type specificity, which determine whether mitochondrial stress promotes immune activation or immune suppression. In general, transient or spatially restricted mitochondrial stress is more likely to promote immune priming through acute interferon signaling and enhanced immune visibility, whereas persistent or unresolved mitochondrial stress is more frequently associated with chronic inflammation, checkpoint activation, metabolic suppression, and immune evasion. Similarly, efficient mitophagic clearance may buffer excessive mitochondrial DAMP signaling, while defective or incomplete mitophagy may amplify mtDNA-driven inflammatory pathways. Importantly, these outcomes are further shaped by cell type, as mitochondrial stress in tumor cells, antigen-presenting cells, myeloid populations, or T lymphocytes may produce distinct immunological consequences.

**Figure 1:**
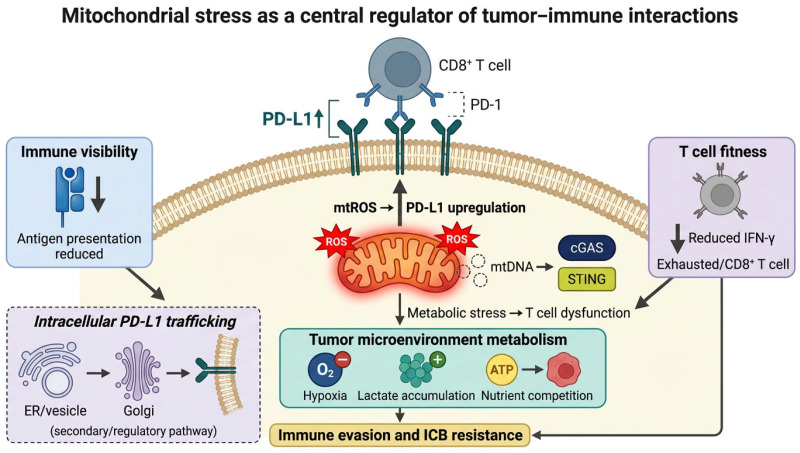
Mitochondrial stress as a central regulator of tumor–immune interactions. Mitochondrial stress in tumor cells and tumor-associated immune cells integrates multiple signaling axes that collectively shape anti-tumor immunity. Canonical PD-L1 is illustrated at the tumor cell plasma membrane, where it interacts with PD-1 on CD8^+^ T cells. At the level of immune visibility, mitochondrial ROS (mtROS) and associated stress responses promote PD-L1 upregulation and impair antigen presentation, reducing tumor recognition by cytotoxic T cells. Mitochondrial DNA (mtDNA) release into the cytosol activates the cGAS–STING signaling pathway, linking mitochondrial stress to innate immune signaling. Concurrently, mitochondrial dysfunction and chronic stress signaling diminish T cell fitness, characterized by reduced interferon-γ (IFN-γ) production and the development of an exhausted CD8^+^ T cell phenotype. At the tumor microenvironment level, metabolic stress contributes to hypoxia, lactate accumulation, and nutrient competition, leading to T cell dysfunction and reduced responsiveness to immune checkpoint blockade (ICB). Intracellular PD-L1 trafficking is illustrated separately as a secondary regulatory pathway to avoid misinterpretation of PD-L1 as a mitochondrial outer membrane protein. These mechanisms collectively contribute to immune evasion and ICB resistance. The schematic illustration was created using graphical tools to summarize key mechanistic interactions. Black arrows indicate signaling direction and pathway interactions, whereas red star symbols represent reactive oxygen species (ROS).

This integrative framework is summarized in Figure 1 and in the graphical abstract, highlighting how mitochondrial stress simultaneously regulates immune visibility, T cell fitness, and tumor microenvironment metabolism to drive immune evasion and limit immunotherapy efficacy. This review focuses on mitochondrial stress as a regulatory axis linking immune evasion to immunotherapy resistance.

## 2. Mitochondrial Stress as an Immune Signaling Hub

Mitochondrial stress responses extend beyond energy production and constitute a regulatory layer shaping immune recognition, effector function, and therapeutic response [11]. In this context, mitochondria can be conceptualized as an immune signaling hub operating through four interconnected layers: (i) mtROS/redox-mediated regulation of transcriptional programs, including hypoxia–inflammation axes; (ii) release of mitochondrial DNA (mtDNA) and activation of innate immune sensors such as cGAS–STING; (iii) metabolic rewiring through OXPHOS–TCA–glycolysis fluxes that shape the metabolite landscape of the tumor microenvironment (TME); and (iv) mitochondrial quality control mechanisms, particularly mitophagy, which determine the magnitude and duration of mitochondrial DAMP signaling [11,16,17]. These layers are interconnected through feedback loops. For instance, hypoxia or electron transport chain (ETC) imbalance increases mtROS production, promoting mtDNA damage and cytosolic release [18]. In parallel, activation of mtDNA–cGAS–STING signaling induces type I interferon programs that modulate checkpoint ligand expression and reshape myeloid cell function in a context-dependent manner [18]. Similarly, tumor-intrinsic oxygen consumption may contribute to hypoxia together with vascular, stromal, and architectural constraints within the tumor microenvironment, thereby amplifying redox signaling and PD-L1–associated immune evasion programs [18]. These processes contribute to variability in response to immune checkpoint blockade (ICB), even among tumors with comparable PD-L1 expression [18]. To improve mechanistic clarity, mitochondrial stress responses can be categorized based on measurable parameters, including signaling dynamics, mtDNA/mtROS levels, and mitophagy efficiency. These parameters collectively determine whether downstream immune outcomes are stimulatory or suppressive.

Within this framework, mitochondrial stress signaling links environmental inputs to immune outcomes. TME stressors such as hypoxia, nutrient deprivation, cytokines, and therapy-induced damage trigger mitochondrial responses that shape antigen presentation, interferon signaling, and immune cell function, ultimately determining sensitivity to checkpoint blockade [7,19,20].

### 2.1. mtROS and Redox Signaling Axis

Mitochondrial reactive oxygen species (mtROS) represent a key signaling component linking mitochondrial dysfunction to immune regulation [18,21]. Hypoxia and ETC imbalance, particularly at complexes I and III, increase electron leakage and mtROS production, acting as both oxidative mediators and signaling regulators such as hypoxia-inducible factor 1α (HIF-1α) and NF-κB [21]. A well-established example of this axis is the direct transcriptional regulation of PD-L1 by HIF-1α under hypoxic conditions, where increased PD-L1 expression in myeloid-derived suppressor cells (MDSCs) suppresses T cell activation [21]. In addition, myeloid-derived suppressor cells (MDSCs) are highly adapted to hypoxic tumor microenvironments and maintain immunosuppressive activity through HIF-1α–dependent signaling pathways [22]. This relationship is functionally causal, as PD-L1 blockade under oxygen-limited conditions restores T cell activation, directly linking redox signaling to immune suppression [21]. In vivo studies further demonstrate that targeting HIF-1α reduces PD-L1-mediated immune evasion within the TME, although such interventions may also affect immune tolerance in normal tissues, highlighting the context dependency of redox-targeted therapies [23].

In addition to HIF-1α, NF-κB signaling represents a key axis linking redox signaling to immune escape [22]. NF-κB regulates PD-L1 expression at both transcriptional and post-transcriptional levels, integrating inflammatory signaling with immune checkpoint control [16,24,25]. A representative mechanism is the TNF-α/NF-κB pathway, where NF-κB–induced CSN5 stabilizes PD-L1 through deubiquitination, thereby enhancing immune evasion and suppressing T cell-mediated cytotoxicity [25]. Notably, the relationship between ROS and PD-L1 expression is context-dependent, as ROS-modulating agents can either increase or decrease PD-L1 levels depending on intersecting pathways such as HIF-1α, NF-κB, and YAP [26]. Together, mtROS-driven activation of HIF-1α and NF-κB converges on PD-L1 regulation, establishing a direct mechanistic link between mitochondrial redox signaling and immune suppression [27,28].

### 2.2. mtDNA Release and Mitochondrial DAMP Biology

Mitochondrial DNA (mtDNA) is a potent danger-associated molecular pattern due to its bacterial origin and CpG-rich structure, rendering it highly immunogenic upon release [17,29]. Its immunological activity depends on its translocation into cytosolic, endosomal, or extracellular compartments, where it engages distinct sensing pathways [29]. Cytosolic mtDNA is primarily detected by the cGAS–STING pathway, which activates type I interferon responses and inflammatory gene programs [30,31,32,33,34]. Early experimental studies have shown that mitochondrial DNA stress, such as that induced by TFAM deficiency, is sufficient to activate innate immune signaling in the absence of infection, establishing a direct link between mitochondrial dysfunction and immune activation [30].

Two major mechanisms of mtDNA release have been characterized. Under sublethal stress conditions, VDAC oligomerization forms pores in the outer mitochondrial membrane, allowing fragmented mtDNA to escape into the cytosol and activate interferon signaling [35]. During apoptosis, BAX/BAK-mediated macropore formation enables mitochondrial herniation and mtDNA release, particularly when caspase activity is impaired, leading to cGAS–STING activation [36]. These findings demonstrate that mtDNA release is not restricted to cell death but can occur in viable cells under stress conditions.

Functionally, mtDNA–cGAS–STING signaling exhibits a dual and context-dependent role in cancer. Acute activation, such as that induced by therapeutic stress, promotes anti-tumor immunity by enhancing antigen presentation and immune priming [19,20,37]. In contrast, chronic activation sustains tumor-promoting inflammatory programs and contributes to metastasis, as demonstrated in models of chromosomal instability [30]. Extracellular mtDNA adds further complexity, as senescent tumor cells release mtDNA that is taken up by myeloid cells and activates cGAS–STING–NF-κB signaling, promoting immunosuppressive phenotypes such as PMN-MDSC expansion [7].

**Figure 2:**
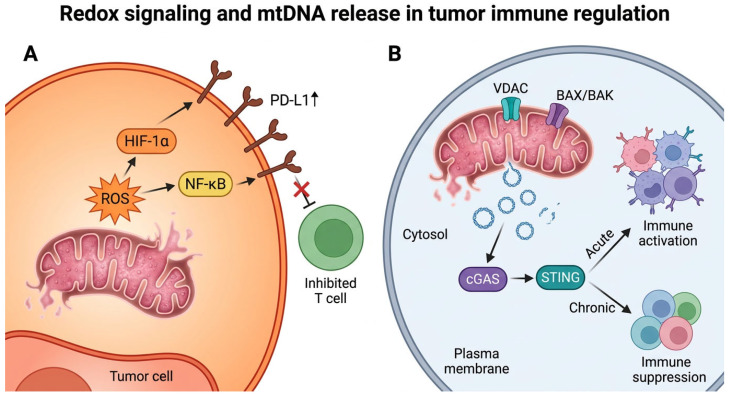
Redox signaling and mitochondrial DNA (mtDNA) release in tumor cells and immune regulation. (**A**) Mitochondrial reactive oxygen species (mtROS) activate redox-sensitive signaling pathways, including HIF-1α and NF-κB, leading to PD-L1 upregulation at the tumor cell plasma membrane and subsequent inhibition of cytotoxic T cell activity. (**B**) Mitochondrial stress promotes mtDNA release from mitochondria into the cytosol through permeability mechanisms involving VDAC and BAX/BAK located on the mitochondrial outer membrane, triggering cytosolic cGAS activation and downstream STING signaling. Acute activation can enhance anti-tumor immunity, whereas chronic activation may contribute to immune suppression and tumor progression. These pathways illustrate how mitochondrial stress-derived signals regulate immune responses in a context-dependent manner. The schematic illustration was created using graphical tools to summarize key mechanisms. Black arrows indicate signaling direction and pathway interactions, whereas red inhibitory symbols indicate suppression of T cell activity.

This duality reflects a central paradox: mitochondrial signals can promote either immune activation or suppression depending on context. Consistently, mtDNA release activates cGAS–STING signaling in a context-dependent manner, where the balance between acute immune activation and chronic immunosuppression determines therapeutic outcome [26,27,38,39]. These redox- and mtDNA-driven signaling pathways are illustrated in Figure 2, highlighting how mitochondrial stress links intracellular damage signals to immune checkpoint regulation and innate immune activation.

### 2.3. Metabolic Reprogramming and Metabolic Competition (OXPHOS–TCA–Glycolysis)

One of the most direct links between mitochondrial stress and immune function is metabolic competition within the TME [36]. Tumor cell OXPHOS activity supports proliferation and contributes to local oxygen depletion together with vascular perfusion limits, stromal organization, and regional microarchitectural constraints within the tumor microenvironment, thereby influencing T cell function [36]. Accordingly, metabolic competition should be interpreted as a context-dependent process shaped not only by tumor metabolic demand, but also by nutrient delivery, oxygen diffusion, perfusion efficiency, and metabolite clearance within the tumor microenvironment. Najjar et al. showed that tumor-intrinsic oxidative metabolism may function as a barrier to PD-1 blockade in melanoma, where high oxygen consumption is associated with increased T cell exhaustion and reduced therapeutic response [36]. These findings suggest that tumor OXPHOS activity shapes TME oxygen tension and constrains checkpoint blockade efficacy through bioenergetic limitations [36].

**Figure 3:**
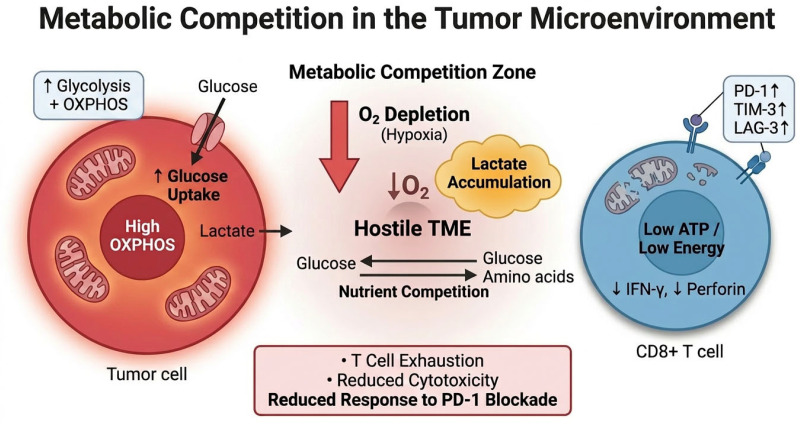
Metabolic competition in the tumor microenvironment (TME) and its impact on anti-tumor immunity. Tumor cells with high glycolytic and oxidative metabolism contribute to local glucose depletion, lactate accumulation, and oxygen limitation together with microenvironmental perfusion and diffusion constraints, establishing a metabolically hostile tumor microenvironment (TME). This environment is characterized by hypoxia, lactate accumulation, and nutrient competition between tumor cells and infiltrating immune cells. As a consequence, CD8^+^ cytotoxic T lymphocytes (CD8^+^ T cells) experience mitochondrial dysfunction and reduced metabolic fitness, leading to impaired effector functions, including decreased interferon-γ (IFN-γ) production and cytotoxic activity. These metabolic constraints promote T cell exhaustion and reduce responsiveness to immune checkpoint blockade, including PD-1–targeted therapies. This metabolic competition model is primarily based on melanoma studies characterized by high tumor-intrinsic oxidative metabolism and oxygen consumption [36]. The red symbol on the CD8^+^ T-cell mitochondrion indicates mitochondrial dysfunction associated with T-cell exhaustion. The schematic illustration was created using graphical tools to summarize key signaling mechanisms. Arrows indicate metabolic and signaling interactions within the tumor microenvironment, whereas red symbols indicate mitochondrial dysfunction and oxygen depletion–associated stress responses.

A second axis of metabolic competition involves lactate accumulation [40]. Tumor-derived lactate suppresses CD8^+^ T cell cytotoxicity by altering metabolic flux and impairing effector function [40]. A third component involves TCA cycle regulation, where inhibition of key enzymes such as PDHA1 and OGDH enhances anti-PD-1 efficacy through ATF3-mediated PD-L1 regulation and metabolic rewiring [37]. These findings indicate that metabolic interventions can induce feedback within immune checkpoint pathways, supporting combination strategies. These metabolic interactions are summarized in Figure 3.

Increased tumor OXPHOS and glycolytic output promote hypoxia and metabolite accumulation, reducing CD8^+^ T cell function, whereas targeting these pathways may restore immune activity in a context-dependent manner [36]. These mechanisms are supported by primary evidence across PD-L1 regulation, mtDNA–cGAS–STING signaling, and metabolic competition, as summarized in Table 1 [19,41].

## 3. Translational Implications: Mitochondria and Immunotherapy Resistance

**Figure 4:**
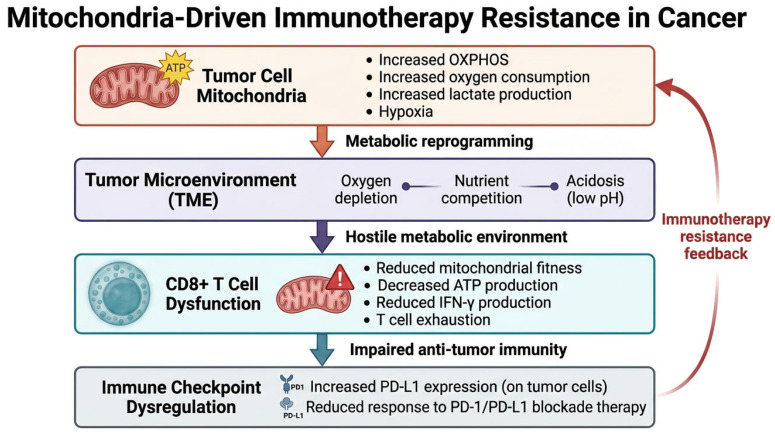
Mitochondria-driven mechanisms of immunotherapy resistance. Tumor-intrinsic mitochondrial metabolism shapes the tumor microenvironment through increased oxygen consumption, lactate production, and metabolic competition. These changes contribute to a hypoxic and nutrient-deprived environment that impairs mitochondrial fitness in tumor-infiltrating lymphocytes, leading to reduced effector function and T cell exhaustion. Concurrently, mitochondrial stress signaling promotes immune checkpoint activation, including PD-L1 upregulation, ultimately reducing responsiveness to immune checkpoint blockade therapies. The illustrated immunotherapy resistance model is primarily based on melanoma studies associated with high oxygen consumption and T-cell exhaustion [36]. The schematic illustration was created using graphical tools to summarize key signaling mechanisms. Arrows indicate the sequential progression of metabolic and immunological interactions contributing to immunotherapy resistance.

Immune checkpoint blockade (ICB), particularly targeting the PD-1/PD-L1 axis, depends on the ability of tumor-reactive T cells to sustain effector function within the tumor microenvironment (TME) [7]. However, this capacity is not determined solely by receptor–ligand interactions but also by mitochondrial function, including energy production, redox balance, and metabolic organization of the TME [7]. As a result, resistance to ICB is increasingly viewed not as a single genetic mechanism but as a phenotype arising from interactions between tumor and immune cell metabolism [7]. The role of mitochondrial metabolism in immunotherapy resistance is illustrated in Figure 4.

Within this framework, mitochondria operate at the center of two major resistance axes. The first, checkpoint resistance, reflects restriction of T cell function through mitochondrial-dependent parameters such as hypoxia and lactate accumulation [36]. The second, metabolic resistance, extends beyond resource competition to include regulation of checkpoint proteins such as PD-L1 and interference with therapeutic antibody activity [36,42].

### 3.1. Mitochondria in Checkpoint Resistance

One of the most direct links between mitochondrial metabolism and ICB resistance arises from tumor-intrinsic oxidative metabolism [36]. In melanoma models, tumors with high oxygen consumption exhibit increased intratumoral hypoxia, enhanced T cell exhaustion, and reduced responsiveness to anti-PD-1 therapy, whereas tumors with reduced oxidative metabolism show selective sensitivity to checkpoint blockade [36]. Clinical data further support an association between elevated oxidative metabolism and disease progression under PD-1 blockade [36].

These findings support a model in which mitochondrial metabolism contributes to tumor microenvironment oxygen tension and T cell functionality in interaction with vascularization, nutrient supply, and tissue architecture [36]. In this context, mitochondria shape the bioenergetic landscape required for effective immune responses, supporting targeting oxygen consumption or hypoxia in combination with ICB [36]. Pharmacological targeting of mitochondrial respiration illustrates the context dependency of this axis. Metformin, a complex I inhibitor, reduces tumor hypoxia and enhances PD-1 blockade efficacy in preclinical models, while also improving T cell survival under hypoxia [43,44]. This dual effect highlights context-dependent mitochondrial mechanisms.

A second major component of checkpoint resistance is the lactate/acidosis axis. Tumor-derived lactate directly suppresses CD8^+^ T cell cytotoxicity by altering metabolic flux, including metabolic signaling pathways [40]. This suppression operates both by limiting effector function within the TME and by reducing the capacity of T cells to recover function even in the presence of PD-1 blockade, thereby creating a metabolic barrier independent of checkpoint signaling [7].

Tumor-mediated suppression of mitochondrial activity within T cells represents an additional resistance mechanism [3]. Certain tumors release soluble factors that inhibit T cell proliferation and mitochondrial function, reducing PD-1 blockade responsiveness [3]. Conversely, pharmacological enhancement of mitochondrial activity, for example with bezafibrate or through PPAR-driven fatty acid oxidation, restores T cell metabolic fitness and improves anti-PD-1 efficacy [3]. Similarly, boosting mitochondrial function through NAD^+^ precursors such as nicotinamide riboside enhances T cell metabolic capacity and therapeutic response [45]. These findings link mitochondrial dysfunction to reduced checkpoint efficacy and identify mitochondrial fitness as a therapeutic target.

### 3.2. Metabolic Resistance and Checkpoint Protein Biology

Metabolic resistance extends beyond TME resource competition to include direct regulation of checkpoint protein biology [42]. Metabolic pathways influence PD-L1 expression, stability, and localization, thereby shaping immune suppression at multiple levels [42].

One well-defined mechanism involves modulation of TCA cycle activity. In melanoma models, inhibition of TCA cycle nodes such as PDHA1 and OGDH enhances anti-PD-1 efficacy by reprogramming glycolysis and regulating PD-L1 expression through ATF3 signaling [37]. These findings illustrate that metabolic interventions frequently induce adaptive feedback, including increased PD-L1 expression, which can be exploited in combination with checkpoint blockade [37].

Post-translational modifications (PTMs) represent a second major layer of regulation [36,42,46,47]. N-glycosylation stabilizes PD-L1 by preventing GSK3β-mediated phosphorylation and β-TrCP-dependent degradation, linking metabolism to checkpoint stability [42]. Similarly, PD-L1 palmitoylation enhances its stability and promotes immune evasion, while inhibition of this modification reduces PD-L1 levels and restores T cell-mediated anti-tumor activity [47]. More recently, metabolite-driven PTMs have been described, including lactate-mediated lysine lactylation, which stabilizes PD-L1 by preventing ubiquitination and degradation, coupling tumor metabolism to checkpoint stability [46].

An additional mechanism of metabolic resistance involves interference with therapeutic antibody binding [48]. In resistant tumors, lactic acid disrupts PD-L1–antibody interactions, reducing therapeutic efficacy, while inhibition of lactate transport (such as MCT-1) restores antibody binding in preclinical models [48].

Metabolic resistance can be conceptualized as a multi-layered structure comprising metabolic competition, tumor-intrinsic rewiring, and PTM-mediated checkpoint regulation [37]. These findings indicate that immunotherapy resistance emerges from converging metabolic, signaling, and proteostatic mechanisms. These mechanisms also define actionable therapeutic targets and associated biomarkers, as summarized in Table 2.

### 3.3. Mitochondria-Based Biomarkers and Combination Strategies

Mitochondrial contributions to ICB resistance provide a framework for identifying biomarkers and combination therapies [36]. Resistance should be interpreted as a composite phenotype characterized by measurable signatures such as hypoxia, lactate production, TIL mitochondrial fitness (including mass, membrane potential, and respiratory reserve), TCA cycle dependencies, and PD-L1 PTM profiles [36].

These converging resistance mechanisms can be operationally categorized into distinct mitochondrial-driven phenotypes with measurable biomarkers and actionable therapeutic strategies [36]. Tumors such as melanoma, which are characterized by high oxygen consumption and hypoxia, may benefit from targeting mitochondrial respiration or hypoxic stress in combination with anti-PD-1 therapy [36]. In lactate-dominant tumor microenvironments, targeting lactate production or transport may help restore T cell function and improve therapeutic efficacy [36]. On the immune side, enhancing mitochondrial fitness through PPAR activation or NAD^+^ supplementation represents a complementary strategy to improve T cell function and treatment response [36,50]. Importantly, these therapeutic strategies differ in their level of validation, ranging from well-established preclinical evidence to emerging experimental observations and concepts that require further clinical investigation. Clear distinction between these levels is essential for translation.

## 4. Mitochondria in Tumor Immune Evasion

Mitochondrial stress responses contribute to tumor immune evasion through multilayered mechanisms that extend beyond immune checkpoint ligand upregulation to include impaired antigen processing and presentation, weakened interferon signaling, loss of mitochondrial fitness in tumor-infiltrating lymphocytes (TILs), and remodeling of the tumor microenvironment (TME) through intercellular organelle transfer [51]. These mechanisms are highly cell-type dependent, as mitochondrial stress in tumor, immune, and stromal cells can produce distinct and sometimes opposing outcomes.

### 4.1. Suppression of Antigen Presentation (MHC-I/IFN-γ Axis)

Low-oxygen conditions are a defining feature of the TME and are associated with immune exclusion and can directly suppress antigen presentation [12,52]. Estephan et al. showed that reduced oxygen availability decreases MHC-I expression in an oxygen-dependent manner through activation of the unfolded protein response (UPR), specifically via the PERK branch, which induces autophagy and targets MHC-I molecules for lysosomal degradation [12,51]. Immunopeptidomics analysis revealed reduced peptide presentation, while pharmacological inhibition of autophagy partially restored antigen presentation [51,52]. These findings indicate that oxygen limitation modulates antigen presentation and immune visibility.

This mechanism indicates that tumor cells evade immune recognition through protein trafficking and degradation pathways [1,51]. Functionally, reduced MHC-I expression correlates with impaired CD8^+^ T cell recognition and infiltration, supporting the concept of an “immune visibility checkpoint” operating within tumors [51]. Mechanistically, intermediates such as SUSD6 and autophagy adaptors like NBR1 have been implicated in linking PERK signaling to selective MHC-I targeting, further refining this axis [51]. From a therapeutic perspective, the reversibility of this process is particularly relevant, as inhibition of autophagy can partially restore MHC-I expression and improve T cell-mediated recognition, supporting combination of autophagy inhibitors with ICB, although systemic effects require consideration [51].

Antigen presentation must also be considered in conjunction with interferon signaling. IFN-γ enhances MHC-I expression but depends on mitochondrial function [53]. Kiritsy et al. showed that mitochondrial respiration is required for IFN-γ responses in antigen-presenting cells (APCs), and that inhibition of complex I impairs APC-mediated T cell activation [52,53]. These findings support a bidirectional dependency between mitochondrial function and antigen presentation in tumor and immune cells [51]. Consequently, strategies aimed at reducing tumor mitochondrial metabolism to alleviate oxygen limitation may simultaneously impair immune cell function, highlighting the need for cell-type–specific therapeutic approaches [51].

A complementary mechanism linking mitochondria to antigen presentation involves direct modulation of mitochondrial electron flow. Mangalhara et al. demonstrated that disruption of complex II enhances tumor immunogenicity by increasing MHC-I expression and antigen-processing gene activation independently of interferon signaling [1,54]. Collectively, these findings position mitochondrial regulation of antigen presentation as a decisive upstream determinant of immune checkpoint blockade efficacy.

### 4.2. Immune Cell Dysfunction (TIL Mitochondrial Fitness and Dynamics)

Tumor immune evasion is also driven by impaired TIL mitochondrial function [51]. Scharping et al. showed that the TME suppresses mitochondrial biogenesis in T cells through downregulation of PGC-1α, leading to metabolic insufficiency and functional impairment in both murine models and human tumors [12,51]. T cells may retain recognition capacity but fail to sustain effector responses due to metabolic insufficiency [12]. Supporting evidence from human tumors shows that CD8^+^ TILs exhibit reduced proliferation and activation capacity accompanied by fragmented mitochondria, altered membrane potential, and dysregulated mtROS levels, indicating that mitochondrial dysfunction independently contributes to T cell impairment [12,55].

PD-1 signaling further reinforces this dysfunction by directly altering mitochondrial structure and function [10,56]. Ogando et al. reported that PD-1 activation reduces mitochondrial cristae density and downregulates structural components such as CHCHD3 and CHCHD10, reducing membrane potential and IFN-γ production [1,56]. In parallel, PD-1 signaling suppresses Drp1-dependent mitochondrial fission, leading to a fused mitochondrial network associated with reduced T cell motility and proliferation, while disrupted mitochondrial dynamics reinforce exhaustion and promote transition to terminally exhausted states [45,51,53].

Importantly, these processes are partially reversible. Supplementation with NAD^+^ precursors such as nicotinamide riboside enhances mitochondrial fitness and improves responses to anti-PD-1 therapy, highlighting mitochondrial restoration as a potential therapeutic strategy [45]. These findings position mitochondrial fitness as a limiting factor for sustained T cell effector function and a key determinant of immunotherapy responsiveness.

### 4.3. TME Remodeling and Organelle Transfer

Mitochondria also contribute to immune evasion through intercellular transfer [57]. Mitochondria can be transferred via tunneling nanotubes or extracellular vesicles, altering metabolic capacity and signaling in recipient cells [57]. Zhang et al. provided evidence for mitochondrial transfer from T cells to tumor cells, demonstrating that this process is associated with enhanced tumor metabolic activity and poor clinical outcomes [55,57]. Conversely, tumor-derived mitochondrial transfer to TILs induces metabolic dysfunction, senescence, and reduced response to PD-1 blockade, while inhibition of extracellular vesicle release partially restores therapeutic sensitivity [15,45].

More recent studies further demonstrate that mitochondrial transfer can reprogram immune signaling. Transfer of mitochondria from immune cells to tumor cells activates mtDNA–cGAS–STING signaling in recipient tumor cells, promoting metastasis and immune evasion [54]. These findings support a model in which the TME functions as an organelle-regulated ecosystem, where mitochondrial exchange reshapes both tumor and immune cell behavior highlighting therapeutic opportunities targeting transfer mechanisms [57].

## 5. Mitophagy and Immune Modulation

Rather than a simple quality control mechanism, mitophagy acts as a regulator of immune signaling thresholds [16,58,59,60]. Mitophagy is a selective lysosomal process that removes damaged mitochondria and represents a component of mitochondrial quality control [58,59,60]. In cancer biology, mitophagy has traditionally been interpreted as a survival mechanism enabling tumor cells to adapt to metabolic stress; from an immunological perspective, mitophagy can be viewed as a rheostat regulating mitochondrial danger-associated molecular patterns (mtDAMPs) [58,59,60]. This dual role highlights that mitophagy cannot be interpreted as inherently immunosuppressive or immunostimulatory, but as a context-dependent process governed by mitochondrial DAMPs, checkpoint turnover, and cell-type–specific responses.

**Figure 5:**
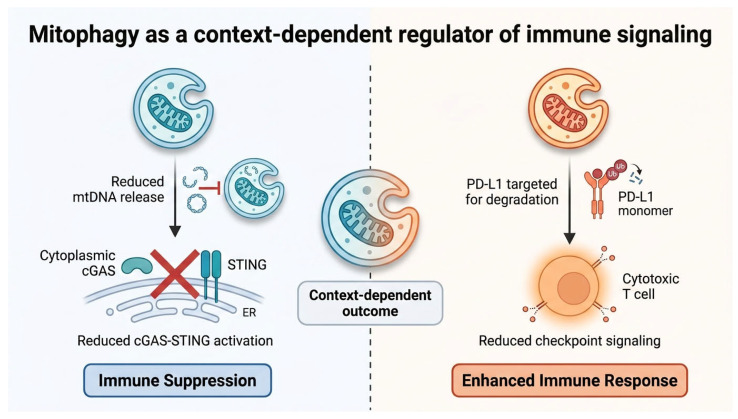
Mitophagy as a context-dependent regulator of tumor immune signaling. Mitophagy plays a dual role in shaping anti-tumor immunity by regulating mitochondrial quality and immunostimulatory signals. On one hand, mitophagy limits mitochondrial DNA (mtDNA) release, thereby reducing cytosolic cGAS activation and downstream STING signaling at the endoplasmic reticulum (ER), suppressing innate immune signaling and contributing to immune evasion. On the other hand, mitophagy can enhance anti-tumor immunity by promoting the degradation of immune checkpoint proteins such as PD-L1 through mitophagy-associated proteostatic mechanisms, leading to reduced checkpoint signaling and enhanced T cell-mediated cytotoxicity. These opposing effects highlight the context-dependent role of mitophagy in tumor–immune interactions. The schematic illustration was created using graphical tools to summarize key mechanisms. Arrows indicate signaling direction and pathway interactions, whereas red inhibitory symbols indicate suppression of cGAS–STING signaling.

Efficient mitophagy limits mtROS accumulation and mtDNA leakage, restraining cGAS–STING signaling and basal inflammation [58,61,62]. In some tumor contexts, this buffering function may reduce immune visibility and facilitate immune escape, whereas in other settings mitophagy may enhance immunotherapy responses by modulating checkpoint protein turnover, including PD-L1 [58]. These effects are illustrated in Figure 5.

The immunological outcome of mitophagy depends on the balance between mitochondrial DAMP clearance, mtDNA compartmentalization, cGAS–STING signaling dynamics, checkpoint protein proteostasis, and cell-type–specific stress responses [58]. In this framework, mitophagy functions as a regulatory rheostat that determines whether mitochondrial stress signals are buffered, amplified, or redirected toward immune activation or immune suppression [58].

### 5.1. Mitophagy as a Regulator of mtDNA–cGAS–STING Signaling

Strong links between mitophagy and immune signaling have been shown in aging models, where mitophagy directly limits mtDNA-driven cGAS–STING activation [58,61,63,64]. Increased cytosolic mtDNA levels correlate with enhanced type I interferon responses, while pharmacological induction of mitophagy, for example with mitophagy-inducing compounds, reduces mtDNA accumulation, cGAS activation, and downstream inflammatory gene expression [31,32,33,58,62,65].

Two implications arise. First, inflammatory output is determined not by absolute mitophagy levels but by the balance between mtDNA release and mitophagic clearance capacity [58,61,66]. Second, mitophagy and cGAS–STING signaling exhibit bidirectional interactions, as inhibition of cGAS signaling can reduce mitophagic activity, indicating the presence of feedback regulation between mitochondrial quality control and innate immune sensing [58]. Evidence from myeloid cells supports this model, where defective mitophagy increases mtDNA leakage and STING activation [66].

### 5.2. Inflammatory Signaling Controls Mitophagy: The TNF Axis

Mitophagy itself is dynamically regulated by inflammatory signals, contributing to mitochondrial–immune crosstalk [67,68]. Tumor necrosis factor (TNF), a key inflammatory cytokine in tumor and inflammatory microenvironments, suppresses PINK1-mediated mitophagy, leading to mitochondrial dysfunction and increased cytosolic mtDNA accumulation [17,67,68]. Mechanistically, TNF-induced mtDNA release activates cGAS–STING signaling through direct mtDNA–cGAS interaction, inducing interferon-stimulated genes [67]. These effects extend to in vivo settings, where cGAS deficiency reduces inflammatory responses and immune cell infiltration in TNF-driven disease models [67].

These findings support a link between inflammation, mitochondrial quality control, and innate immune activation. Within the TME, inflammatory cues may shift mitophagy toward dysfunctional clearance, amplifying mtDNA–STING signaling [69]. Importantly, restoring mitophagy does not uniformly suppress anti-tumor immunity; instead, it may normalize aberrant, chronic DNA sensing and re-establish balanced inflammatory signaling depending on context [69].

### 5.3. Mitophagy and Immune Checkpoint Regulation: PD-L1 Proteostasis

Mitophagy regulates immune checkpoint biology through PD-L1 localization and degradation. In triple-negative breast cancer, mitochondrial localization of PD-L1 is associated with improved response to chemo-immunotherapy, whereas membrane-localized PD-L1 correlates with poor clinical outcomes [25,42,49]. Mechanistically, PINK1-dependent recruitment of PD-L1 to mitochondria promotes its degradation via mitophagy, while ATAD3A inhibits this redistribution and stabilizes PD-L1 at the cell membrane [49].

Chemotherapeutic agents such as paclitaxel can increase ATAD3A expression, suppress mitophagy, and promote PD-L1 accumulation, suggesting a mechanism of therapy resistance [49]. These findings highlight two implications: PD-L1 subcellular localization may serve as a predictive biomarker, and targeting the ATAD3A–PINK1–mitophagy axis may enhance immunotherapy efficacy by promoting PD-L1 degradation [49].

Additional evidence from glioblastoma models further supports this relationship. OMA1-driven mitochondrial dysfunction induces mitophagy while simultaneously increasing mtDNA release and activating cGAS–STING signaling, resulting in elevated PD-L1 expression and immune evasion [25,70]. This demonstrates that incomplete or dysregulated mitophagy may paradoxically enhance checkpoint activation, emphasizing mitophagic efficiency rather than absolute activity.

### 5.4. Mitophagy Defects and Tumor Immune Escape

Disruption of mitophagy contributes to immune evasion. Loss of Parkin, a key ubiquitin ligase, is associated with impaired antigen presentation, reduced CD8^+^ T cell infiltration, and resistance to immunotherapy [71,72,73]. Clinical data further demonstrate that reduced Parkin expression correlates with poorer survival and diminished therapeutic response, supporting a link between mitophagy deficiency and immune escape [71].

### 5.5. Reliability Considerations in Mitophagy–STING Literature

Interpretation of the mitophagy–STING axis requires careful consideration of methodological variability across experimental systems. Mechanistic conclusions should rely on reproducible evidence across multiple models [31,32,33,58,61,62,66,67,69].

A framework integrates three lines of evidence: (i) mitophagy induction reduces mtDNA–cGAS–STING activation, (ii) mitophagy impairment increases mtDNA leakage and STING signaling, and (iii) inflammatory signals such as TNF suppress mitophagy and enhance mtDNA-driven immune activation [31,32,33,58,61,62,66,67,69]. This triangulated model provides a structured framework for understanding mitophagy–immune interactions in cancer.

### 5.6. Mechanistic Summary

**Figure 6:**
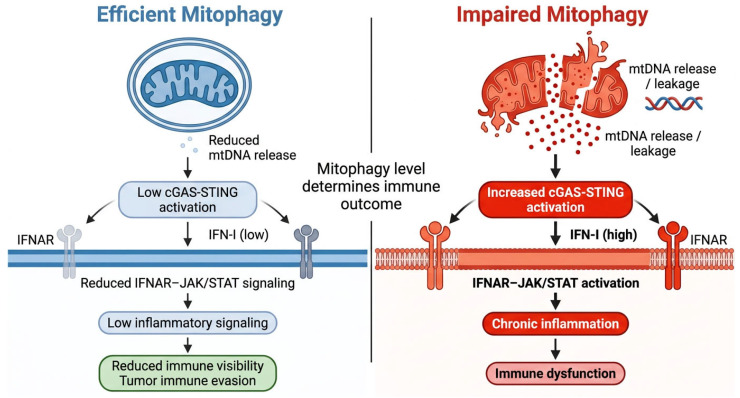
Mitophagy as a context-dependent regulator of mitochondrial danger-associated signaling. Efficient mitophagy limits mitochondrial DNA (mtDNA) release and suppresses cytosolic cGAS–STING activation, thereby reducing downstream IFNAR–JAK/STAT signaling and decreasing immune visibility. In contrast, impaired or insufficient mitophagy promotes mtDNA accumulation and leakage, leading to increased cGAS–STING activation and sustained IFNAR–JAK/STAT activation through type I interferon signaling, which may either support anti-tumor immunity under acute conditions or drive chronic inflammation and immune dysfunction depending on context. These opposing effects position mitophagy as a regulatory rheostat controlling mitochondrial DAMP signaling and downstream immune outcomes. The schematic illustration was created using graphical tools to summarize key signaling mechanisms. Arrows indicate signaling direction and pathway interactions, whereas red symbols represent enhanced inflammatory and cGAS–STING–associated signaling responses.

Conceptually, the net immune effect of mitophagy reflects the balance between mitochondrial DAMP clearance, mtDNA–cGAS–STING activation, PD-L1 proteostasis, and cell-type–specific stress responses [58,61,62,66,67,69]. Efficient mitophagic flux may limit excessive inflammatory signaling by preventing mtDNA accumulation, whereas defective or incomplete mitophagy may enhance chronic STING activation and immune dysregulation [58,61,62,66,67,69]. In certain contexts, mitophagy can promote anti-tumor immunity by directing PD-L1 to mitochondria for degradation, reducing checkpoint-mediated suppression [58,61,62,66,67,69]. Inflammatory signals such as TNF suppress mitophagy, leading to increased mtDNA release and activation of DNA sensing pathways with context-dependent immune outcomes [58,61,62,66,67,69]. Therefore, mitophagy should be interpreted as a context-dependent regulator of immune signaling thresholds rather than a unidirectional pro- or anti-tumor mechanism. These roles are summarized in Figure 6.

## 6. Experimental Models to Study Mitochondrial–Immune Interactions

Understanding how mitochondrial stress regulates immune evasion requires systems capturing metabolic gradients, organelle signaling, and immune function [74]. While reductionist models provide mechanistic clarity, 3D and in vivo systems are required to recapitulate physiological constraints [74].

### 6.1. Role and Limitations of 2D Culture Systems

Two-dimensional (2D) monolayer cultures remain indispensable for mechanistic studies, including genetic perturbations, pharmacological screening, and time-resolved signaling analyses [74]. Their primary advantage is reduced complexity, enabling dissection of causal relationships between mitochondrial pathways and immune-relevant signaling nodes [74]. However, key determinants, including oxygen and nutrient gradients, extracellular matrix composition, and multicellular interactions, are poorly represented in 2D systems [74]. Consequently, mitochondrial processes that depend on microenvironmental constraints, such as oxygen limitation–driven autophagy–MHC-I regulation or metabolic competition, cannot be modeled in monolayer cultures [51].

### 6.2. 3D Spheroid Models: Capturing Gradient Biology

Three-dimensional tumor spheroids provide an intermediate platform that captures features of avascular tumor regions, including spatial gradients of oxygen, nutrients, and metabolites, as well as cell–cell interactions and diffusion-limited drug penetration [75]. As spheroids increase in size, they develop distinct zones, including proliferative outer layers, quiescent intermediate regions, and hypoxic or necrotic cores, resembling in vivo tumor architecture [75]. This gradient formation is relevant for studying mitochondrial stress responses, as oxygen limitation within spheroid cores induces metabolic rewiring, DNA damage responses, and suppression of antigen presentation [76].

Importantly, key mechanisms described in earlier sections, including PERK–autophagy-mediated MHC-I degradation and reduced immunopeptidome diversity, can be reproduced in 3D systems but are largely absent in 2D cultures [51]. Recent ultrastructural studies using 3D endometrial cancer spheroids have demonstrated that lithium-induced mitochondrial stress leads to organelle remodeling and non-apoptotic growth suppression, supporting mitochondrial stress as a driver of tumor adaptation [77].

Despite these advantages, spheroid systems present technical challenges, including size heterogeneity and the need for careful standardization [74]. In addition, most spheroid models lack endogenous immune components, limiting their ability to fully recapitulate immune–tumor interactions [74].

### 6.3. Patient-Derived Organoids: Genetic and Structural Fidelity

Patient-derived organoids (PDOs) represent a more advanced platform that preserves tumor genetic heterogeneity, histological features, and aspects of tissue architecture more faithfully than cell line–based systems [78]. These models are suitable for studying tumor-intrinsic mitochondrial mechanisms, including antigen presentation, metabolic rewiring, and stress signaling pathways.

Two complementary strategies are used in organoid-based immuno-oncology studies [79]. Reconstruction systems involve the addition of immune cells, such as peripheral blood mononuclear cells (PBMCs), tumor-infiltrating lymphocytes (TILs), or antigen-specific T cells, enabling controlled investigation of immune–tumor interactions [79]. In contrast, air–liquid interface (ALI) systems preserve endogenous immune components within the organoid structure, enabling more physiologically relevant modeling of tumor immune microenvironments and checkpoint blockade responses [80]. Selection between these approaches depends on the experimental question, as tumor-intrinsic mitochondrial mechanisms can be studied in epithelial organoids, whereas immune response dynamics require immune-competent systems [1].

### 6.4. Organoid–Immune Co-Culture Systems

Organoid–immune co-culture platforms represent a powerful approach for linking mitochondrial mechanisms to functional immune outcomes [81]. These systems allow simultaneous assessment of clinically relevant endpoints, including generation of tumor-reactive T cells, quantitative measurement of tumor cell killing, and evaluation of immune checkpoint blockade responses ex vivo [81]. Co-culture of patient-derived tumor organoids with autologous lymphocytes enables enrichment of tumor-reactive T cells and direct measurement of cytotoxic activity, providing a readout of immune competence [81].

These platforms are suited for testing hypotheses derived from mitochondrial biology, such as whether modulation of mitochondrial electron transport enhances antigen presentation and T cell-mediated killing, or whether metabolic interventions alter sensitivity to checkpoint blockade.

### 6.5. Mitochondria-Focused Readouts Linking Mechanism to Immune Function

To experimentally validate mitochondrial–immune interactions, mitochondrial function must be assessed alongside immune outcomes [51]. Bioenergetic profiling, including measurements of oxygen consumption rate (OCR) and extracellular acidification rate (ECAR), reflects the balance between OXPHOS and glycolysis and reflects tumor oxygen consumption, a key determinant of oxygen-limited immune suppression and checkpoint resistance [82]. At the cellular level, assessment of mitochondrial ROS, membrane potential, and mitochondrial mass reflects mitochondrial fitness, particularly in TILs, where dysfunction is linked to exhaustion and impaired immunotherapy response [75,82].

Mitochondrial DNA release represents a critical mechanistic bridge between mitochondrial stress and innate immune activation [17]. Quantification of cytosolic or extracellular mtDNA enables assessment of DAMP signaling, particularly when interpreted alongside markers of cell death to distinguish active release from passive leakage [17]. Downstream signaling can be evaluated through activation of the cGAS–STING pathway, including phosphorylation of STING, TBK1, and IRF3, as well as interferon-stimulated gene expression [51]. Given the context-dependent nature of mtDNA signaling, experimental design should distinguish between acute and chronic activation windows, as these can produce fundamentally different immune outcomes [17].

Finally, antigen presentation and functional immune responses must be incorporated as endpoint measurements [51]. Surface MHC-I expression and immunopeptidomics provide direct measures of immune visibility, while T cell cytotoxicity assays, cytokine production, and degranulation link mitochondrial perturbations to functional immune outcomes. Integration of these readouts establishes causality between mitochondrial stress, antigen presentation, and T cell-mediated killing [51].

### 6.6. Integration with In Vivo Models

Although in vitro systems provide high mechanistic resolution, in vivo tumor models remain essential for capturing systemic immune dynamics and vascularization [83]. In murine models, suppression of mitochondrial biogenesis within T cells drives metabolic insufficiency and functional impairment of tumor-infiltrating lymphocytes, limiting response to immune checkpoint blockade [83]. Similarly, tumor-intrinsic metabolic features, including high oxygen consumption, contribute to hypoxic microenvironments that restrict T cell function and reduce responsiveness to PD-1 blockade, effects that can be partially reversed through metabolic targeting [36].

These findings highlight that reductionist and 3D systems are indispensable for mechanistic discovery, in vivo models are required to validate how mitochondrial stress responses shape immune evasion and therapeutic resistance under physiologically relevant conditions.

## 7. Future Directions

Mitochondria-centered immune regulation can now be translated into quantitative and clinically testable frameworks [29]. Three priorities emerge: defining acute versus chronic mtDNA–STING signaling, developing a unified tumor “visibility score,” and testing these mechanisms in patient-specific 3D platforms [17,29,51].

### 7.1. Quantifying Acute vs. Chronic mtDNA–STING Signaling

A central challenge is that mtDNA–cGAS–STING signaling can promote both anti-tumor immunity and immune suppression, indicating that its biological impact is quantitatively regulated [17,29,34]. Immune outcomes depend on mtDNA dose, duration, localization, and cell type [29]. These observations indicate threshold-dependent rather than binary signaling [29].

Mechanistically, distinct modes of mtDNA release generate different signaling dynamics [1]. VDAC oligomerization enables controlled mtDNA leakage, whereas BAX/BAK macropore formation during apoptosis leads to large-scale mitochondrial herniation and rapid mtDNA efflux [1]. In parallel, caspase activation suppresses downstream inflammatory signaling, shaping STING output [1].

Clinical observations support these distinctions. Senescent tumor cells release extracellular mtDNA that activates cGAS–STING–NF-κB signaling in myeloid cells, promoting PMN-MDSC-mediated immunosuppression and tumor progression [29]. In addition, mitochondrial transfer from immune cells to tumor cells can trigger mtDNA-driven STING activation and type I interferon signaling, supporting metastasis and immune escape [13,34,84].

These findings suggest that chronic STING activation may favor immune evasion. Together, these observations support a threshold-based model in which mitochondrial stress responses transition from immune-stimulatory to immune-suppressive states depending on signaling magnitude, persistence, compartmentalization, and mitophagic buffering capacity. Future work should therefore define quantitative thresholds corresponding to (i) acute activation associated with immune priming, (ii) chronic activation associated with myeloid reprogramming and suppression, and (iii) a mitophagy buffering threshold that determines whether mtDNA release is contained or amplified [29]. These observations also raise the theoretical possibility that controlled delivery of exogenous mtDNA could be used to activate innate immune signaling. However, such approaches remain highly context dependent, as excessive or chronic mtDNA exposure may promote immunosuppressive signaling and tumor progression rather than anti-tumor immunity.

### 7.2. Integrating Mitochondrial Metabolism with Tumor Visibility

A second priority is developing a “tumor visibility score” integrating antigen presentation, mitochondrial metabolism, and interferon signaling [1,51,52]. Here, the “tumor visibility score” is defined as the capacity of tumor cells to present antigens and sustain interferon signaling in a manner that enables immune detection. Evidence shows that mitochondrial processes regulate immune recognition. Reduced oxygen availability suppresses antigen presentation through PERK–UPR-mediated autophagy, leading to MHC-I degradation and reduced immunopeptidome diversity [51], while mitochondrial respiration is required for effective IFN-γ signaling in antigen-presenting cells, linking bioenergetics to immune activation capacity [52].

Conversely, modulation of mitochondrial electron flow can enhance antigen presentation independently of interferon signaling, indicating that mitochondrial metabolism exerts direct control over tumor immunogenicity [1]. Tumor visibility can be conceptualized as a multidimensional parameter that includes MHC-I surface expression, immunopeptidome diversity, IFN-γ signaling competence, and mitochondrial metabolic state, including oxygen consumption and hypoxia stress [85].

This framework addresses limitations of biomarkers such as PD-L1 or T cell-inflamed gene expression signatures, which may fail to capture metabolic or antigen presentation–driven immune escape [85]. For example, tumors with preserved interferon signaling may still evade immune responses through high oxygen consumption and metabolic competition, as demonstrated in melanoma models, limiting T cell function despite apparent immune activation signatures [36]. Future studies should validate this composite visibility score in patient-derived systems and correlate it with responses to immune checkpoint blockade [86].

### 7.3. Patient-Specific 3D Platforms and Multi-Omic Perturbation

The third priority is testing mitochondrial–immune mechanisms in patient-specific systems [40]. Organoid–immune co-culture models enable the generation of tumor-reactive T cells and direct measurement of tumor cell killing, providing readouts of immune competence [40].

Recent advances demonstrate that CRISPR-based genetic perturbation can be integrated into these systems to identify tumor-intrinsic regulators of immune sensitivity [86]. Genome-wide CRISPR screens in organoid–T cell co-culture systems have identified key determinants of immune-mediated tumor killing, while CRISPR perturbation combined with single-cell transcriptomics enables high-resolution mapping of gene–phenotype relationships [86].

Future studies should integrate genetic perturbation with multi-omic readouts, including single-cell transcriptomics, metabolomics, and immunopeptidomics, to establish causal links between mitochondrial function, antigen presentation, and immune response [87]. These approaches will allow systematic mapping of the pathway from mitochondrial stress to immune phenotype and therapeutic outcome [87].

### 7.4. Standardization and Translational Integration

Methodological standardization is essential for reproducibility and clinical relevance [88]. Temporal dynamics of pathways such as cGAS–STING must be defined, distinguishing acute from chronic activation using time-resolved measurements of phospho-STING, TBK1, and interferon responses [29]. Omics-based readouts, including immunopeptidomics, require standardized quality control metrics such as false discovery rate thresholds and reproducibility criteria compatible with clinical workflows [52].

In addition, experimental parameters in organoid–immune co-culture systems, including T cell source, effector-to-target ratios, and organoid size distribution, must be consistently reported, as these variables directly influence derived metrics such as tumor visibility and immune response [89]. Finally, integration of mitochondrial biomarkers with established clinical predictors, including T cell-inflamed gene expression profiles and tumor mutational burden, is necessary to translate these frameworks into predictive tools [85].

## 8. Conclusions

Mitochondria have emerged as key regulators of tumor–immune interactions, extending beyond their classical bioenergetic roles to coordinate immune signaling, antigen presentation, and microenvironmental dynamics. In this review, we propose that mitochondrial stress responses function as a unifying axis governing immune visibility, immune cell fitness, and the metabolic architecture of the tumor microenvironment. In addition, mitochondria-derived signals such as reactive oxygen species and mitochondrial DNA dynamically regulate innate immune pathways, including cGAS–STING, with context-dependent consequences ranging from immune activation to immunosuppression.

Within this framework, mitophagy introduces an additional regulatory layer by controlling mitochondrial quality and modulating the release of immunostimulatory signals. Its function is inherently bidirectional, as it can dampen immune activation by limiting mtDNA release while also enhancing immunotherapy efficacy through regulation of checkpoint protein turnover, including PD-L1. These observations support a model in which mitochondrial stress responses orchestrate a multi-layered network underlying immune evasion and therapeutic resistance.

From a translational perspective, targeting mitochondrial metabolism, mtDNA sensing, and mitophagy offers complementary strategies to enhance immune checkpoint therapies. Future advances will depend on integrating these approaches in physiologically relevant 3D and patient-derived models. In this context, mitochondrial stress may be considered not only a consequence of tumor progression but also a determinant of immune fate and therapeutic response. Importantly, the functional consequences of mitochondrial stress are not unidirectional but are shaped by signaling dynamics, cellular context, and the balance between mitochondrial damage and compensatory quality control pathways. Collectively, these observations support a context-dependent model in which mitochondrial stress responses dynamically shift between immune-stimulatory and immune-suppressive states according to signaling intensity, duration, compartmentalization, and mitophagic buffering capacity.

## Figures and Tables

**Table 1:** Representative primary studies supporting mitochondrial stress-mediated regulation of tumor–immune interactions.

Axis	Model/Approach	Mechanistic Insight	Immune Outcome	Therapeutic Implication	Reference
Hypoxia–PD-L1	Tumor-bearing mice; MDSCs; hypoxia	HIF-1α directly regulates PD-L1 transcription under hypoxia	Suppression of T cell activation	Rationale for HIF/PD-L1 targeting combinations	[22]
HIF targeting	In vivo TME models	HIF-1α targeting reduces PD-L1-mediated immune evasion	Restoration of anti-tumor immunity	Combination potential with ICB	[21]
mtDNA release (VDAC)	Cell-based assays; pore formation	VDAC oligomers enable mtDNA fragment release into cytosol	Activation of type I IFN signaling	Targeting mtDNA–STING dynamics	[26]
Apoptotic mtDNA release	Apoptosis models; caspase inhibition	BAX/BAK macropores facilitate mtDNA efflux	cGAS–STING activation and IFN response	Immunogenic cell death modulation	[28]
mtDNA stress signaling	TFAM deficiency; innate immune assays	mtDNA stress activates ISG programs	Enhanced innate immune signaling	Controlled DAMP-based activation strategies	[27]
Senescence–mtDNA	Therapy-induced senescence; PMN-MDSC	Extracellular mtDNA activates cGAS–STING–NF-κB in myeloid cells	Increased immunosuppression	Targeting senescence-associated mtDNA signaling	[29]
OXPHOS barrier	Melanoma; patient + murine models	High oxygen consumption limits T cell function	Increased T cell exhaustion	OXPHOS targeting + ICB combinations	[36]
Lactate suppression	Metabolic tracing; CD8 assays	Lactate alters pyruvate flux and succinate signaling	Reduced CD8^+^ cytotoxicity	Targeting lactate metabolism	[40]
TCA targeting	Melanoma; PDHA1/OGDH inhibition	TCA disruption modulates PD-L1 via ATF3	Enhanced response to anti-PD-1	Metabolic + ICB combination strategies	[37]

**Table 2:** Mitochondria-targeted therapeutic strategies, associated biomarkers, evidence levels, and translational limitations in tumor immunotherapy.

Target	Mechanism	Example Approach	Main Cell Type Affected	Evidence Level	Biomarker(s)	Key Limitation/Risk	Reference
OXPHOS	Reduces tumor oxygen consumption	Metformin + anti-PD-1	Tumor cells/T cells	Primarily preclinical with emerging clinical evidence	Tumor hypoxia (HIF-1α), oxygen consumption rate (OCR)	Systemic metabolic effects and context-dependent effects on immune cells	[36,43,44]
mtDNA–cGAS–STING	Context-dependent activation of innate immune signaling	STING pathway modulation	Tumor cells/APCs/myeloid cells	Preclinical	Cytosolic mtDNA, p-STING, p-TBK1, IFN-stimulated genes (ISGs)	Chronic STING activation may promote immunosuppression and inflammatory toxicity	[19,20]
Mitophagy	Modulates PD-L1 turnover and mitochondrial immune signaling	PINK1/ATAD3A axis	Tumor cells	Preclinical	PINK1/Parkin levels, LC3-II, PD-L1 subcellular localization	Strong context- and cell-type dependency of mitophagy responses	[49]
Lactate metabolism	May improve T cell function in the TME	MCT inhibition	Tumor cells/CD8^+^ T cells	Preclinical	Lactate levels, MCT1 expression, extracellular acidification rate (ECAR)	Metabolic compensation pathways may reduce therapeutic efficacy	[40,48]
T cell mitochondrial fitness	Enhances T cell metabolic capacity	NAD^+^ supplementation	T cells	Emerging preclinical evidence	Mitochondrial mass, membrane potential (ΔΨm), PGC-1α expression	Limited clinical validation and durability data	[45]

## Data Availability

No new data were created or analyzed in this study.

## Abbreviations

The following abbreviations are used in this manuscript:

TME	Tumor microenvironment
ICB	Immune checkpoint blockade
mtROS	Mitochondrial reactive oxygen species
mtDNA	Mitochondrial DNA
cGAS	Cyclic GMP-AMP synthase
STING	Stimulator of interferon genes
OXPHOS	Oxidative phosphorylation
TCA	Tricarboxylic acid cycle
PD-1	Programmed cell death protein 1
PD-L1	Programmed death-ligand 1
IFN-γ	Interferon gamma
TILs	Tumor-infiltrating lymphocytes
MHC-I	Major histocompatibility complex class I
PERK	Protein kinase RNA-like endoplasmic reticulum kinase
UPR	Unfolded protein response
MDSCs	Myeloid-derived suppressor cells
FAO	Fatty acid oxidation
CD8	Cluster of Differentiation 8
